# Impact of Mammalian Two-Pore Channel Inhibitors on Long-Distance Electrical Signals in the Characean Macroalga *Nitellopsis obtusa* and the Early Terrestrial Liverwort *Marchantia polymorpha*

**DOI:** 10.3390/plants10040647

**Published:** 2021-03-29

**Authors:** Mateusz Koselski, Vilmantas Pupkis, Kenji Hashimoto, Indre Lapeikaite, Agnieszka Hanaka, Piotr Wasko, Egle Plukaite, Kazuyuki Kuchitsu, Vilma Kisnieriene, Kazimierz Trebacz

**Affiliations:** 1 Department of Plant Physiology and Biophysics, Faculty of Biology and Biotechnology, Institute of Biological Sciences, Maria Curie-Sklodowska University, Akademicka 19, 20-033 Lublin, Poland; mateusz.koselski@poczta.umcs.lublin.pl (M.K.); agnieszka.hanaka@poczta.umcs.lublin.pl (A.H.); piotr.wasko@poczta.umcs.lublin.pl (P.W.); 2 Department of Neurobiology and Biophysics, Institute of Biosciences, Life Sciences Center, Vilnius University, Sauletekio av. 7, LT-10257 Vilnius, Lithuania; vilmantas.pupkis@gmc.vu.lt (V.P.); indre.lapeikaite@gf.vu.lt (I.L.); egle.plukaite@gmc.stud.vu.lt (E.P.); 3 Department of Applied Biological Science, Tokyo University of Science, Noda, Chiba 278-8510, Japan; kenji.hashimoto@rs.tus.ac.jp (K.H.); kuchitsu@rs.tus.ac.jp (K.K.)

**Keywords:** plant action potential, SV/TPC channels, verapamil, tetrandrine, NED-19

## Abstract

Inhibitors of human two-pore channels (TPC1 and TPC2), i.e., verapamil, tetrandrine, and NED-19, are promising medicines used in treatment of serious diseases. In the present study, the impact of these substances on action potentials (APs) and vacuolar channel activity was examined in the aquatic characean algae *Nitellopsis obtusa* and in the terrestrial liverwort *Marchantia polymorpha*. In both plant species, verapamil (20–300 µM) caused reduction of AP amplitudes, indicating impaired Ca^2+^ transport. In *N. obtusa*, it depolarized the AP excitation threshold and resting potential and prolonged AP duration. In isolated vacuoles of *M. polymorpha*, verapamil caused a reduction of the open probability of slow vacuolar SV/TPC channels but had almost no effect on K^+^ channels in the tonoplast of *N. obtusa.* In both species, tetrandrine (20–100 µM) evoked a pleiotropic effect*:* reduction of resting potential and AP amplitudes and prolongation of AP repolarization phases, especially in *M. polymorpha,* but it did not alter vacuolar SV/TPC activity. NED-19 (75 µM) caused both specific and unspecific effects on *N. obtusa* APs. In *M. polymorpha*, NED-19 increased the duration of repolarization. However, no inhibition of SV/TPC channels was observed in *Marchantia* vacuoles, but an increase in open probability and channel flickering. The results indicate an effect on Ca^2+^ -permeable channels governing plant excitation.

## 1. Introduction

Mammalian cells, including human cells, harbor two genes *hTPC1* and *hTPC2* encoding two-pore-Na^+^-selective ion channels located in endosomes and lysosomes, respectively [1]. Recently, these endomembrane ion channels have attracted the attention of researchers since they are involved in many severe diseases including Ebola virus infection [2], neoangiogenesis in several types of tumors, arrhythmias, etc. [3].

In plants, slow-vacuolar (SV) cation channels are located in the tonoplast, i.e., the vacuolar membrane and exhibit slow kinetics of activation. They were among the first channels discovered in plant cells long before characterization of their animal counterparts [4]. Since that time, these channels have been characterized with the patch clamp technique in all tested plant species except characean algae (due to difficulties of vacuole isolation).

Plant cells also possess two-pore channels (TPCs). Many plant species, including model vascular plants *Arabidopsis thaliana* and *Oryza sativa* (rice), have only one gene encoding TPCs. *Arabidopsis thaliana AtTPC1* [5] was shown to be localized in the tonoplast and identified as encoding the SV channel [6]. Rice *OsTPC1* was suggested to encode the plasma membrane Ca^2+^-permeable channel [7,8] in addition to the SV channel in the vacuolar membrane [9]. Rice and tobacco TPC channels have been suggested to play a role in defense and oxidative stress signaling [10,11,12]. 

Recently, two independent research teams have published the crystallographic structure of this channel derived from vacuoles of *A. thaliana* [13,14]. Electrophysiological, genetic, and crystallographic studies have revealed that SV/TPC in plant vacuoles is a Ca^2+^- and voltage-dependent, nonselective cation channel permeable to both mono- and divalent cations [15,16,17]. In addition to the slow kinetics of its activation (hundreds of ms), it strongly rectifies allowing cation flow mainly from the cytoplasm to the vacuole lumen. The activity of the channel is regulated by many natural and artificial factors that prevent uncontrolled ion leakage in steady state conditions [15]. However, its Ca^2+^ dependence seems to be the most important trait from a physiological point of view. It is activated by a high cytoplasmic Ca^2+^ concentration with [Ca^2+^]_cyt_ exceeding 10 µM [4]. A high luminal Ca^2+^ concentration suppresses the channel activity [18,19]. Ca^2+^-dependent regulation, and especially the Ca^2+^ permeability of the SV/TPC channel, has opened a discussion about its physiological role. The permeability of the SV/TPC channel to calcium ions gives rise to the idea of so-called “calcium-induced-calcium-release” (CICR)—an increase in [Ca^2+^]_cyt_ owing to Ca^2+^ influx through the plasma membrane activates SV/TPC channels, amplifying the calcium signal and exceeding its threshold to initiate cascades of physiological responses [20]. The problem is that the channels rectify in a “wrong” direction, allowing Ca^2+^ flux to the vacuolar lumen rather than to the cytosol, and require a high nonphysiological [Ca^2+^]_cyt_ for their activation. That is why many researchers were skeptic about this concept [21,22]. Quite recently, new light has been shed on this idea. It was demonstrated that the velocity of propagation of calcium waves and concomitant long-distance electrical signals evoked by different stimuli drastically decreased in *tpc1*^KO^ mutants in respect to the wild type [23,24], pointing to the role of SV/TPC channels in long-distance signaling in plants.

It was documented that drugs such as NED-19, tetrandrine, and verapamil that are targeted to human TPC channels have positive therapeutic effects against several serious diseases [14]. In the present study, we examined the impact of these compounds on long-distance electrical signals in phylogenetically ancient plants: a characean alga (*Nitellopsis obtusa*) and a liverwort (*Marchantia polymorpha*). *Marchantia polymorpha* represents liverworts, which are regarded as pioneer terrestrial plants that colonized lands c.a. 500 million years ago, whereas aquatic *Nitellopsis obtusa* belongs to charophyta algae—direct ancestors of land plants [25]. Both these species are excitable, i.e., they are able to generate action potentials (APs). In response to local stimuli, APs spread throughout the thalli of *M. polymorpha* gametophytes [26] or along giant internodal cells of *N. obtusa* [27]. Ca^2+^ plays the central role in initiation of APs and regulation of the activity of ion channels involved in AP generation. Investigations of electrical signaling in a single *N. obtusa* cell and multicellular *M. polymorpha* upon exposure to drugs targeting Ca^2+^-permeable channels, especially SV/TPC, may indicate the role of these channels in long-distance signaling.

In the present study, we examined the effects of verapamil, NED-19, and tetrandrine on the electrogenesis of APs and the rate of their transmission. Additionally, using the patch clamp technique, we examined direct effects of these compounds on SV/TPC channel activity in isolated vacuoles of *M. polymorpha* and on K^+^-permeable channels in cytoplasmic droplets of *N. obtusa*. Since mammalian TPC channels are located in endomembranes (endosomes and lysosomes), their isolation and patch clamp examination are strongly limited. Thus, one of the aims of this study was to test a possibility of establishing a low-cost plant-based platform to examine the effectiveness of different drugs targeted to human TPC channels.

## 2. Results

### 2.1. Action Potentials in Nitellopsis obtusa Cells

300 µM verapamil (*n* = 6) significantly depolarized membrane resting potential from −210 ± 11 mV in APW (artificial pond water, control solution) to −140 ± 62 mV (*p* = 0.017). AP excitation threshold potential *E_th_* was depolarized from −101 ± 12 mV to −73 ± 35 mV (*p* = 0.047). Both AP peak potential *V_max_* and AP amplitude *A_th_* were unaffected by verapamil; however, AP amplitude *A_RP_* was significantly decreased from 254 ± 14 mV to 178 ± 79 mV (*p* = 0.044). It was observed that verapamil prolonged AP depolarization duration *t_dep_* (from 0.8 ± 0.1 s to 1.6 ± 0.7 s, *p* = 0.016) and repolarization duration *t_rep_* (from 2.6 ± 0.7 s to 8 ± 8.6 s, *p* = 0.031). Thus, the whole AP duration *t_1/2_* was also significantly prolonged (from 3.5 ± 0.8 s to 9.6 ± 9.2 s, *p* = 0.031).

**Figure 1:**
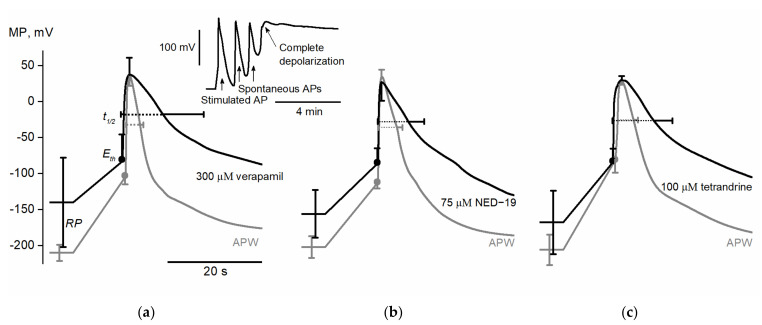
Average electrically evoked action potentials of *Nitellopsis obtusa* internodal cells in artificial pond water (APW) with standard conditions and after exposure to different inhibitors of human two-pore channels (TPCs): (**a**) APs after exposure to 300 µM verapamil. The upper insert represents spontaneous APs after electrical excitation (indicated by arrows) and subsequent complete depolarization of membrane potential; (**b**) APs after exposure to 75 µM NED-19; (**c**) APs after exposure to 100 µM tetrandrine. Horizontal bars indicate SD of *t_dep_* and *t_rep_*; vertical—resting potential (*RP*), excitation threshold (*E_th_*), and peak potential *V_max_* for each drug *n* = 6−7.

We conclude that verapamil depolarizes membrane resting potential and AP excitation threshold potential while prolonging AP duration. 300 µM verapamil often (in 10 cells out of 12) caused complete and irreversible membrane depolarization which, coupled with cessation of cytoplasmic streaming and loss of turgor, was considered as cell death (Figure 1). Besides the observed electrophysiological effects, which can be explained by the influence of verapamil on ion transport systems, its effect on cell viability suggests that the substance influences other processes not necessarily related to ion transport and excitation.

75 µM NED-19 (*n* = 7) significantly depolarized membrane resting potential from −201 ± 15 mV in APW to −155 ± 33 mV (*p* = 0.007). The drug also depolarized AP excitation threshold potential *E_th_* (from −108 ± 9 mV to −81 ± 20 mV, *p* = 0.012). The AP peak value *V_max_* remained stable, but NED-19 decreased both AP amplitude *A_th_* (from 143 ± 10 mV to 107 ± 31 mV, *p* = 0.012) and AP amplitude *A_RP_* (from 231 ± 17 mV to 181 ± 33mV, *p* = 0.002). The AP depolarization phase was unaffected, and only a trend toward prolongation of the repolarization phase was observed (*t_rep_* in APW lasted 3.5 ± 1.2 s and 6 ± 3.4 s after exposure (*p* = 0.078)). Nevertheless, the total AP duration *t_1/2_* was significantly prolonged from 4.4 ± 1.2 s to 6.9 ± 3.4 s (*p* = 0.037).

We conclude that NED-19 acts in a similar manner to verapamil: it depolarizes membrane resting potential and AP excitation threshold potential, and thus decreases AP amplitude while prolonging AP duration. Treatment with 75 µM NED-19 was lethal to only one cell in eight.

100 µM tetrandrine (*n* = 7) significantly depolarized membrane resting potential from −203 ± 21 mV in APW to −165 ± 44 mV (*p* = 0.031). Neither AP excitation threshold *E_th_*, peak value *V_max_* nor AP amplitude *A_th_* were significantly affected, but a trend was seen of AP amplitude *A_RP_* to decrease (*A_RP_* value in APW 233 ± 21 mV compared to 197 ± 42 mV after the exposure to tetrandrine (*p* = 0.063)). Tetrandrine prolonged AP depolarization duration *t_dep_* (from 1.3 ± 0.2 s to 1.9 ± 0.3 s, *p* = 0.035) and repolarization duration *t_rep_* (from 2.6 ± 0.8 s to 8.2 ± 3.9 s, *p* = 0.023). Thus, the whole AP duration *t_1/2_* was also significantly prolonged from 3.9 ± 0.9 s to 10.1 ± 4 s (*p* = 0.02).

We conclude that exposure to 100 µM tetrandrine affects several of the selected parameters, namely membrane resting potential and AP duration, in a similar manner as the exposure to verapamil and NED-19. The preliminary experiments with 200 µM tetrandrine indicated that the drug may depolarize AP excitation threshold *E_th_* and thus decrease AP amplitude *A_Eth_* in a similar manner to verapamil and NED-19.

The alterations of *RP* and AP parameters in *N. obtusa* are summarized in Table 1. The average APs under the exposure to the tested drugs are summarized in Figure 1.

### 2.2. Action Potential Conduction Velocity Along an Internodal N. obtusa Cell

During AP conduction velocity evaluation experiments, the whole cell was exposed to a particular solution. Registration began immediately after a 10-min incubation period. The registration lasted for 3–4 h unless the cell became unexcitable sooner.

In APW, the AP conduction velocity was 2.4 ± 0.8 cm/s (*n* = 12). 100 µM verapamil did not affect AP conduction velocity (2.5 ± 1.2 cm/s, *n* = 5), but verapamil-induced spontaneous APs were conducted significantly more slowly compared to the control (0.6 ± 0.1 cm/s, *n* = 7, *p* < 0.001). In the verapamil solution, spontaneous APs were also conducted more slowly than electrically induced APs (*p* = 0.026). Spontaneous activity was induced in all treated cells and they eventually (after ~1 h) became unexcitable and lost turgor (*n* = 17), which was treated as cell death.

75 µM NED-19 did not significantly decrease the AP conduction velocity (1.8 ± 0.5 cm/s, *n* = 6, *p* = 0.082). No prominent spontaneous activity was observed; however, four out of the six treated cells eventually (after ~2 h) became unexcitable.

200 µM tetrandrine significantly decreased the AP conduction velocity compared to its velocity in APW (1.4 ± 0.2 cm/s, *n* = 6, *p* < 0.001). 200 µM tetrandrine-induced spontaneous APs were conducted significantly more slowly compared to the stimulated APs in APW (0.5 ± 0.1 cm/s, *n* = 4, *p* = 0.03) and in 200 µM tetrandrine (electrical stimulation) (*p* < 0.001). Eventually (after ~2.5 h), six out of the seven cells treated with tetrandrine became unexcitable.

**Figure 2:**
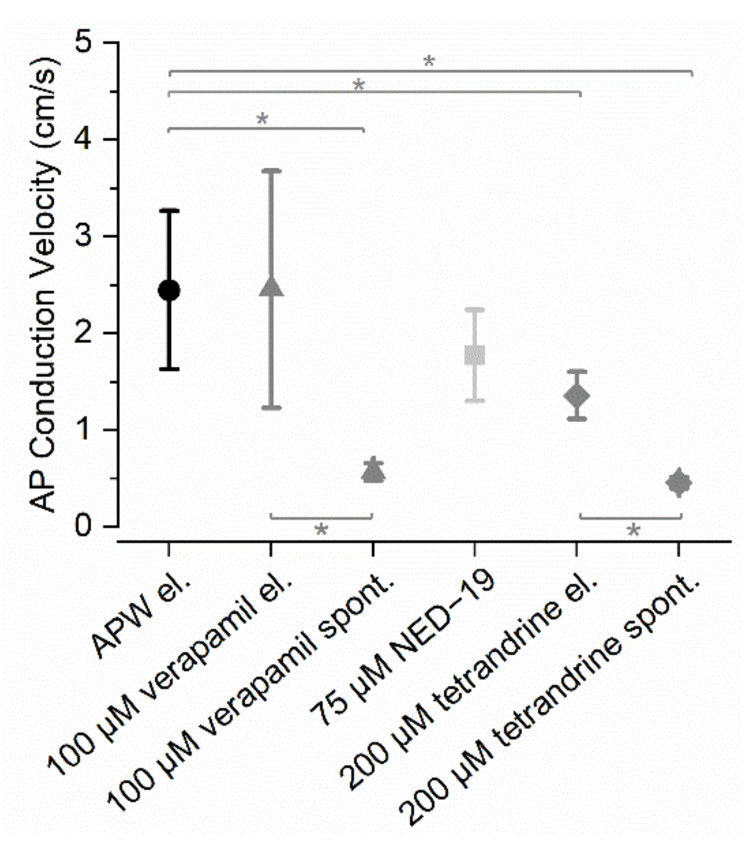
Average AP conduction velocities in *N. obtusa* in the standard solution APW and after exposure to the drugs: 100 µM verapamil, 75 µM NED-19, and 200 µM tetrandrine. APs were stimulated electrically unless a drug evoked a spontaneous AP train. Asterisks indicate significant differences, *n* = 4−12.

The AP conduction velocities after exposure of *Nitellopsis obtusa* to the drugs are compared in Figure 2.

### 2.3. Patch Clamp Measurements on N. obtusa Cytoplasmic Droplet Membrane

In the *N. obtusa* cytoplasmic droplet membrane (which is composed of the tonoplast) in artificial cell sap (ACS), the 70 pS K^+^ channel activity was the most prominent (*n* = 9), though it was rarely possible to observe the activity of smaller conductance ion channels.

We observed that 300 µM verapamil (*n* = 7) significantly increased the current amplitude in voltages more negative than −60 mV ((*p* = 0.011 (−80 mV), *p* = 0.018 (−100 mV), *p* = 0.02 (−120 mV)). While the 70 pS channel open probabilities were highly variable over the whole voltage range, verapamil significantly decreased the open probability in the voltage range from −20 mV to −80 mV (*p* = 0.045 (−20 mV), *p* = 0.022 (−40 mV), *p* = 0.022 (−60 mV) and *p* = 0.02 (−80 mV)).

75 µM NED-19 (*n* = 8) did not affect either the current amplitude or the channel open probability.

**Figure 3:**
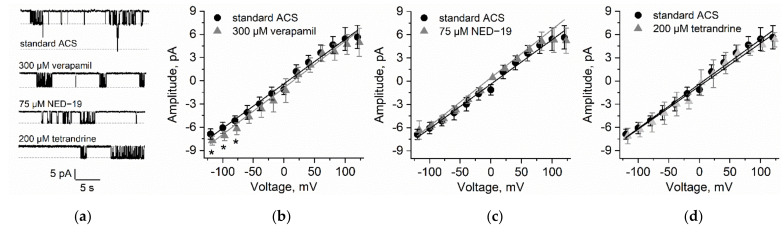
Comparison of high-conductance K^+^-permeable channel activity in the tonoplast of *N. obtusa* in the control solution ACS and after application of 300 µM verapamil, 75 µM NED-19, and 200 µM tetrandrine. (**a**) Examples of current traces obtained at −80 mV. Dashed lines indicate open states. The measurements were prepared in the tonoplast-attached configuration. Single channel I/V curves obtained after exposure to 300 µM verapamil (**b**), 75 µM NED-19 (**c**), and 200 µM tetrandrine (**d**). Asterisks indicate significant differences, *n* = 5−9.

200 µM tetrandrine (*n* = 5) did not affect current passing through the 70 pS channel amplitudes, though it increased the channel open probabilities at −40 mV (*p* = 0.046) and −100 mV(*p* = 0.048) (Figure 3).

### 2.4. Action Potentials in Marchantia polymorpha Cells

**Figure 4:**
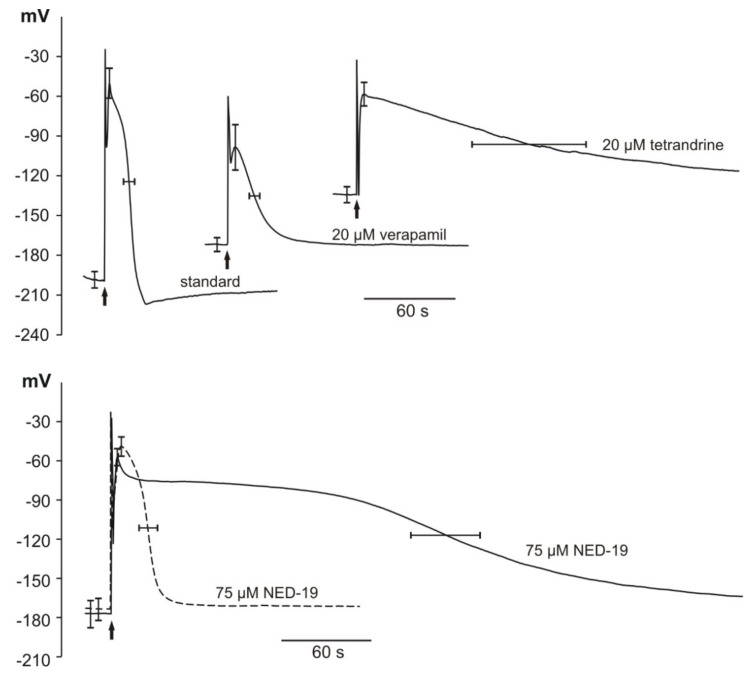
Influence of different inhibitors of human TPCs on electrically evoked action potentials in *Marchantia polymorpha*. Representative intracellular recordings observed in the standard solution and the standard solution supplemented with different inhibitors. Arrows indicate the moment of stimulation (4 V for 1 s). Stimulation evoked an artifact which is visible at the beginning of each recording. Two kinds of responses evoked by NED-19-short (dashed lines) and long lasting (solid lines) are presented at the bottom of the figure. The values of membrane potential are presented on vertical axes.

The microelectrode measurements indicated that the application of electrical stimulation evoked APs in the *Marchantia polymorpha* cells. The resting membrane potential (*RP*) amounted to −187 ± 12 mV (*n* = 16). The responses observed in the cells in the vicinity of the cathode (one of the stimulating electrodes) were recorded after the application of rectangular voltage pulses 4 V lasting 1 s. At the moment of stimulation, a stimulation artifact appeared (in the shape of sharp spikes visible in Figure 4); next, the AP was recorded. In the control (untreated plants), the amplitude of AP (*A_RP_*) reached 139 ± 19 mV (*n* = 16) and the duration measured in the half of the amplitude (*t_1/2_*) amounted to 24.7 ± 7.3 s (*n* = 16). The maximal membrane potential at the peak of the AP (*V_max_*) equaled −47 ± 23 mV (*n* = 16). The time required to reach the *V_max_* (depolarization time, *t_dep_*) was 4.8 ± 1.6 s (*n* = 16) and the time of repolarization measured in the half of the amplitude (*t_rep_*) was 19.9 ± 6.5 s (*n* = 16).

Subsequently, we treated *Marchantia* plants with verapamil, tetrandrine, and NED-19. Verapamil (20 µM) caused a statistically significant shift of *RP* to more positive values (−163 ± 10 mV, *n* = 10) and *V_max_* to more negative values (−98 ± 34 mV, *n* = 6). Verapamil also caused a significant reduction of *A_RP_* to 70 ± 33 mV (*n* = 6), which is consistent with its well-known ability to block Ca^2+^-permeable channels. The higher concentrations of verapamil of 100 µM and 50 µM caused permanent depolarization of the *Marchantia* cells and rendered them unresponsive to the stimulation.

In comparison to APs recorded in the standard solution, the responses after the tetrandrine treatment (20 µM) started from more positive *RP* (−130 ± 12 mV, *n* = 8) and were characterized by significantly lower amplitude (72 ± 13 mV, *n* = 8). Moreover, the tetrandrine treatment caused substantial prolongation of the repolarization phase of APs to 169.8 ± 76.2 s (*n* = 7), compared to 19.9 ± 6.5 s (*n* = 16) in the control plants. Tetrandrine applied in the higher concentration (40 μM) caused permanent depolarization of the membrane potential.

Statistically significant changes in the parameters describing APs in *Marchantia* were also obtained after the application of NED-19. The responses to NED-19 differed in the duration; hence, we decided to divide the results into two groups: APs with short duration (*t_1/2_* in this group was in the range from 23.4 s to 61 s) and long duration (*t_1/2_* in the range from 131.4 s to 235.2 s). The *RP* was reduced to 92.2% and 88.4% for the short- and long-duration groups, respectively. The same trend was observed in the case of the AP amplitudes—reduction to 90.6% and 85.6% for the short- and long-lasting APs, respectively. The most striking difference was observed in the duration of the repolarization phase in the long-duration group in comparison to the control (prolongation by 925%). NED-19, like verapamil and tetrandrine, caused irreversible depolarization of *M. polymorpha* cells when applied at the higher concentration (100 μM).

The parameters describing resting and action potentials in *M. polymorpha* cells are summarized in Table 2. and examples of traces of action potentials are presented in Figure 4.

### 2.5. Patch Clamp Measurements on Vacuoles Isolated from Marchantia polymorpha Cells

**Figure 5:**
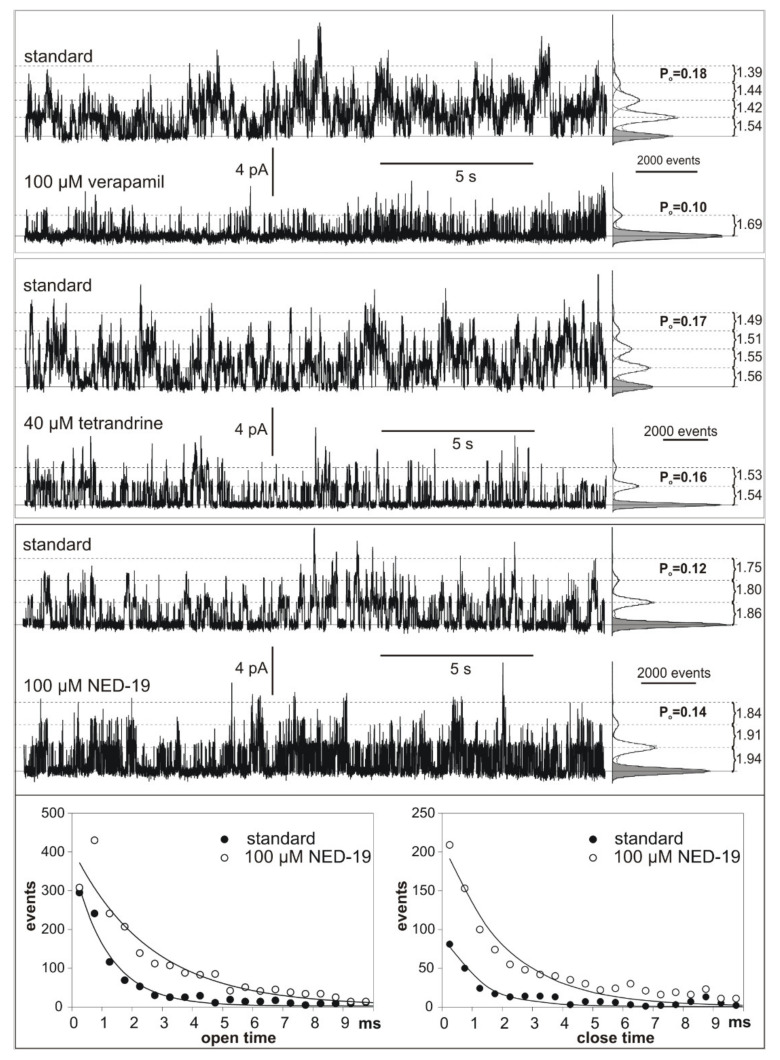
Comparison of SV/TPC channel activity recorded after the application of 100 µM verapamil, 40 µM tetrandrine, and 100 µM NED-19. The traces were obtained at 60 mV. The solid line and grey histograms indicate the closed state, and the dashed line and white histograms indicate open states. Amplitudes of the currents flowing through the single channels and open probabilities (P_o_) obtained from the histograms are indicated. The measurements were prepared in the cytoplasm-out configuration.

SV channels in vacuoles isolated from *M. polymorpha* cells were characterized using the patch clamp technique in one of our previous studies [28]. In the present study, we applied solutions on both sides of the tonoplast patches, which activate SV channels and exclude activation of any other known ion channels in the vacuolar membrane [29] (see Materials and Methods 4.3.4). The SV/TPC channel activity of *M. polymorpha* vacuoles was tested in terms of their dependence on different chemicals: 100 µM verapamil, 40 µM tetrandrine, and 100 µM NED-19. None of the inhibitors completely blocked the SV/TPC channel activity, but a reduction of the open probability was observed, especially after the treatment with verapamil (from 0.18 to 0.1). Almost no reduction of the open probability was observed after the application of tetrandrine (from 0.17 to 0.16). Each of the chemicals also caused a reduction of the number of active channels in the patch. Examples of the effects of verapamil, tetrandrine, and NED-19 on single SV/TPC currents are presented in Figure 5. NED-19 evoked an increase in the open probability (from 0.12 to 0.14) and flickering of the channels was observed. After the NED-19 administration, open and close times (especially shorter than 10 ms) were observed more frequently than in the nontreated vacuoles (Figure 5).

## 3. Discussion

The ability of the plant to generate long-distance signals—action potentials (APs)—was preserved upon transition from the aquatic to terrestrial environment, although the “menu” of stimuli changed dramatically. Plants that possess basal positions in a process of terrestrialization, namely characean fresh water macroalgae and liverworts, or, more generally, bryophytes, have been examined for years in this aspect with application of different electrophysiological techniques. As a result of this investigation, an ion mechanism of APs was proposed, first for characean algae [30], then adopted for liverworts [31] and for vascular plants [32,33]. According to this scheme, the AP is initiated with Ca^2+^ influx through the plasma membrane followed by activation of Ca^2+^-dependent Cl^−^-permeable channels, which both are responsible for the depolarization phase of the AP. Repolarization is regarded to be an effect of K^+^ efflux and subsequent activation of the electrogenic proton pump, H^+^-ATPase. Participation of Ca^2+^ pools from internal stores (vacuoles, ER) in the initial phase of AP was postulated in characean algae and liverworts. In internodal cells of algae, potential changes across the tonoplast were registered together with APs across the plasma membrane, suggesting coupling of these phenomena.

The mechanism of AP generation across the tonoplast seems to be similar; the depolarization phase is governed by Ca^2+^ and Cl^−^ channel activity, whereas repolarization is caused by increased tonoplast permeability to K^+^ [30,34]. The main difference between APs of these two plant groups—characean algae and liverworts—is their kinetics. The duration of APs in the algae is in a range of seconds, whereas APs in liverworts last dozens of seconds. In this parameter, liverworts resemble most of the vascular plants examined so far, except specialized plants such as *Dionaea*, *Mimosa*, etc.

The main question concerning APs in plants is: what is their molecular basis, i.e., which ion channel proteins are responsible for AP generation and propagation? Deciphering of the genomes of the characean alga *Chara braunii* [35] and the liverwort *Marchantia polymorpha* [36] was a milestone in solving this problem. While obtaining *M. polymorpha* mutants with knocked-out ion channel genes is feasible, the same task in the case of the alga seems difficult due to the multinuclear structure of their cells. This justifies the electrophysiological approach, as it may provide new facts concerning the nature of APs, which can be considered at the molecular level in the future.

Mammalian TPCs differ substantially from those in plants, including *Marchantia.* They are Na^+^-selective and activated by a low-abundance lipid, i.e., phosphatidylinositol-3,5-bisphosphate PI(3,5)P_2_, and probably indirectly by nicotinic acid adenine dinucleotide phosphate NAADP. No Ca^2+^ dependence of these channels has been reported. These physiological differences between plant and human TPCs occur irrespective of the strong structural similarities. Since human TPC1 and TPC2 are involved in the etiology of many serious diseases, we examined in plant cells the effects of pharmaceuticals that yield positive therapeutic effects, assuming that molecular mechanisms underlying channel regulation may be common. We have chosen *N. obtusa* and *M. polymorpha* as the objects of our study, as these plants are good examples of adaptation to environmental changes, from water to land conditions. We expected that the variability of processes regulating ion channels, which are primary sensors of environmental changes, would help to find similarities in pharmacology with human TPCs.

Verapamil has been applied to plant cells before. It was demonstrated to affect Ca^2+^ transport and Ca^2+^-based signaling. For instance, in the liverwort *Conocephalum conicum*, closely related to *Marchantia polymorpha*, 200 µM verapamil caused reduction of the amplitudes of light-induced voltage transients (VTs) to 45% ± 9% of the control [37]. VT is regarded as a Ca^2+^ component of APs in this liverwort [38,39]. Verapamil also reduced the amplitudes of cold-induced responses in *Arabidopsis thaliana*, *Helianthus annuus*, and *Vicia faba* [39]. 

In line with those results, the investigation of *N. obtusa* revealed the depolarizing effect of verapamil on the AP excitation threshold (*E_th_*) (Figure 1). The precise mechanism implicating positive feedback during the acute mobilization of Ca^2+^ to the cytoplasm during excitation in plants is unclear. Still, in principle, the membrane potential of excitation is determined by the voltage dependency of Ca^2+^-dependent Cl^−^ channels and their regulation by [Ca^2+^]_cyt_ [40,41]. The verapamil-induced lag of depolarization coincides with depolarized *E_th_*. Similar effects, indicating calcium signaling impairment, were recorded under exposure to Ni^2+^ [42]. The activation of Ca^2+^ permeable channels of *N. obtusa* resulted in hyperpolarized *E_th_* [43].

In *Marchantia*, we observed strong reduction (by approximately 50%) of AP amplitudes, which indicates reduction of Ca^2+^ fluxes. Verapamil is a membrane-permeable compound, which may affect both plasma membrane- and endomembrane Ca^2+^-permeable channels. Patch clamp experiments on isolated vacuoles of *M. polymorpha* revealed reduction of SV channel activity. However, in this case, Na^+^ instead of Ca^2+^ were used as the main permeating ions, and the observed reduction in the SV channel open probability was mainly attributed to the reduction of Na^+^ fluxes to the vacuole lumen. It can be assumed that verapamil had a similar reducing effect on Ca^2+^ fluxes across the tonoplast, as SV is a nonselective cation-permeable channel. Probably, both Ca^2+^ fluxes, i.e., through the plasma membrane and the tonoplast, were reduced by verapamil to such an extent that putative Ca^2+^-dependent Cl^-^-permeable ion channels responsible for AP depolarization were only partially activated by the low level of [Ca^2+^]_cyt_. Verapamil only slightly affected the resting membrane potential and the repolarization phase of APs, which indicates that the electrogenic H^+^-ATPase responsible for the resting potential and the K^+^ outward rectifying channels participating in AP repolarization were only partially suppressed.

Conversely, depolarization of membrane resting potential and a decrease in the AP amplitude *A_RP_* were observed in *N. obtusa*. Tsutsui and coworkers [44] reported a similar effect after exposure of *Chara corallina* cells to 100 µM verapamil. They also observed a decrease in membrane resting conductance, linking the combined effect to inhibition of H^+^-ATPase. Prolongation of repolarization can be attributed to inhibition of Ca^2+^-ATPase activity. Ca^2+^-ATPase governs Ca^2+^ resequestration to inner sources after excitation, and thus reduces the activity of Ca^2+^-dependent Cl^−^ channels [45]. A blocking effect of verapamil on K^+^ channels cannot be excluded either. In most cases, the exposure to verapamil was lethal, thus underlining its nonspecific effect on *N. obtusa*.

Tetrandrine is a bis-benzylisoquinoline alkaloid isolated from the plant *Stephania tetrandrea* [14]. Tetrandrine (20 µM) had pleiotropic effects on membrane- and action potentials in *M. polymorpha*. It caused depolarization of the resting potential from −188 mV to −130 mV. Similar to verapamil, tetrandrine reduced the AP amplitude, but in contrast to verapamil, it caused prolongation of the repolarization phase of APs by a factor of approximately ten. All these effects point to a lack of specificity of tetrandrine in *Marchantia* cells. It seems to affect Ca^2+^- and K^+^-permeable channels and the proton pump in the plasma membrane. No significant effect of this compound on SV/TPC channel activity in the vacuole was observed. 

The exposure to tetrandrine affected *N. obtusa* in a similar manner: the resting membrane potential was depolarized and the AP depolarization and repolarization phases were prolonged. Thus, the effect was also similar to the effect of verapamil on *N. obtusa*. However, tetrandrine did not affect the AP excitation threshold potential or the AP amplitude. Since preliminary experiments revealed that a higher concentration of tetrandrine (200 µM vs 100 µM) did depolarize *E_th_*, this might indicate that the nonspecific effect of tetrandrine (possibly affecting H^+^- or Ca^2+^-ATPases or K^+^ channels) is more potent than the specific effect.

In mammalian cells, NED-19 acts as a noncompetitive inhibitor of NAADP-activated TPC channels in endolysosomes [14]. In *Arabidopsis thaliana*, NAADP does not regulate the SV/TPC channel [46]. In our experiments, *Marchantia* cells responding to NED-19 can be divided into two groups: no significant effect on duration of APs was registered in the first and substantial prolongation of the repolarization phase of APs occurred in the other. It can be assumed that the tip of the microelectrode in the first case was located in the cytosol, whereas the tip was located in the vacuole lumen in the other case. Our long-lasting experience indicates that, in untreated *M. polymorpha* cells, there is no significant difference in the transmembrane potential regardless of the localization of the microelectrode tip in the cytosol or in the vacuole. The same concerns the shape of APs. The localization of the microelectrode tip in different cell compartments is stochastic, which was confirmed in the liverwort *Conocephalum conicum* cells impaled with H^+^-selective microelectrodes [47]. Two groups of results were obtained, one with pH 7.16 attributed to the cytosol localization and 5.83 when the microelectrode tip was localized in the vacuolar compartment [47]. Assuming that in *Marchantia*, like in characean cells, the tonoplast contributes to APs, one can imagine that NED-19 causes prolongation of the vacuolar component of the AP exerting its effect on vacuolar conductivity, and the effect is observed only if the microelectrode tip is inserted into the vacuole. The patch clamp data on isolated *M. polymorpha* vacuoles showed that NED-19 indeed affects vacuolar ion currents. However, instead of the expected blockage of SV/TPC channels, it causes flickering—high-frequency opening and closing events. SV/TPC1 channels can contribute to excitation, as demonstrated by Jaslan et al. [48], who recorded long-lasting vacuolar potential changes in wild-type *Arabidopsis* but not in the TPC1 loss-of-function mutant *tpc1-2*.

All AP parameters of *N. obtusa* were distributed unimodally because the electrode tip was always located in the vacuole (determinable by the RP, peak value, and shape of AP) [45]. Similar to verapamil, NED-19 depolarized the membrane excitation threshold *E_th_*, leading to decreased AP amplitude *A_Eth_.* Depolarization of the membrane resting potential upon exposure also resulted in lesser A_RP_. Another nonspecific effect included prolongation of AP duration *t_1/2_*. Interestingly, while all three drugs exhibited similar specific and nonspecific effect patterns, NED-19 exerted the most potent effect.

As shown in patch clamp investigations, K^+^ conductance dominates in the characean tonoplast. Considerable attention has been paid to high-conductance (~170 pS) K^+^ channels in the tonoplast of *Chara corallina* [49,50]. In *Chara gymnophylla*, existence of smaller conductance K^+^ permeable channels (~90 pS) has been reported as well. These channels exhibit relatively high permeability to Na^+^ compared with K^+^ ions (0.4) [51]. In the *N. obtusa* cytoplasmic droplet membrane, only lower conductance (~80 pS) channel activity was observed. Na^+^ permeability was reported to be 0.2. Ca^2+^ activates these channels, but the permeability of neither Ca^2+^ nor other divalent cations is known [52].

The AP propagation velocity in a single *N. obtusa* cell was investigated via the extracellular electrode technique. Out of the three tested drugs, the exposure to verapamil and tetrandrine led to trains of spontaneous APs. We observed that the drug-induced spontaneous APs in *N. obtusa* were conducted significantly more slowly than the electrically induced APs. While the electrically induced APs were induced at 10-min periods, spontaneous APs were generated at much higher frequencies; at the start of a spontaneous AP train, the period was usually 1−2 min. Such a short inter-AP period implies that the cell did not reach its steady pre-excitation state, i.e., the cytoplasmic Ca^2+^ concentration was not reduced to steady state levels. This assumption is supported by preliminary experiments, which revealed that electrical stimulation at a higher frequency decreases the AP propagation velocity in *N. obtusa*.

Verapamil, tetrandrine, and NED-19 targeted as pharmaceuticals to two-pore channels (TPC1 and TPC2) in the endomembranes of human cells affect plant ion channels in a nonspecific way. *N. obtusa* and *M. polymorpha* exhibited different susceptibilities to the applied chemicals. Hence, different concentrations were tested in a range between causing no effect and affecting cell viability. 

The reduction of the AP amplitudes by verapamil and tetrandrine in the algae *Nitellopsis obtusa* and in the liverwort *Marchantia polymorpha* indicates inhibition of Ca^2+^ fluxes responsible for the depolarization phase of APs in the plants. The possibility to determine the excitation threshold and its alterations in *Nitellopsis obtusa* cells allows linking the effect of NED-19 and verapamil to the Ca^2+^-dependent initiation of excitation. The prolongation of the AP repolarization phase by tetrandrine and NED-19 indicates suppressed outward-rectifying K^+^ channels participating in AP repolarization or Ca^2+^-ATPase inhibition. The patch clamp studies revealed no significant differences in the vacuolar ion channel activities in both species, except the verapamil effect in *M. polymorpha*. It can be concluded that the pharmacology of vacuolar ion channels in the evolutionarily ancient plant species differ significantly from that in mammalian TPC1 and TPC2 channels. Moreover, our results indicate that these substances affect different ion channels in plant cells, which opens a question of their specificity as pharmaceuticals due to possible side effects.

## 4. Materials and Methods

### 4.1. Plant Material and Tested Drugs

Mature giant algae *Nitellopsis obtusa* (N.A. Desvaux) J. Groves were collected from Lithuanian lakes during autumn months and maintained in aquariums as described in [53]. Prior to the experiments, the internodal cells (second or third below the tip) were separated from neighboring internodal cells and kept at least overnight in buffered artificial pond water (APW) in daylight conditions.

Control solutions for *N. obtusa*: artificial pond water (APW) (in mM: 0.1 KCl, 1.0 NaCl, 0.1 CaCl_2_, and 2.5 Tris, adjusted to pH 7.2 by Hepes); artificial cell sap (ACS) (in mM: 100 KCl, 10 NaCl, 10 CaCl_2_, 10 MgCl_2_, adjusted to pH 5.3 by Mes). Tested drugs: verapamil (100 µM or 300 µM), trans-NED-19 (75 µM, solved in 0.13% DMSO in APW); tetrandrine (100 µM, solved in 0.75% DMSO in APW (for investigations of AP parameters) or 200 µM, solved in 1.5% DMSO in APW (for investigations of AP conduction velocity and tonoplast ion channel activity)). All solutions were maintained in pH and ion concentrations as APW or ACS.

Liverworts *Marchantia polymorpha* (Tak-1 wild-type) were kept in closed Petri dishes filled with solid agar medium in a growing chamber (Conviron Adaptis A1000, Winnipeg, Canada). The plants grew at a temperature of 23 °C, 60 µmol/m^2^ s light intensity, and 16/8 light/dark conditions. Four- to six-week-old plants were used in the electrophysiological studies.

Two to five hours before the experiments, fragments of the *Marchantia* thallus were mounted in experimental chambers filled with a bath solution under 60 µmol/m^2^ s light. The standard bath solution contained (in mM): 1 KCl, 0.1 CaCl_2_, 50 sorbitol, 2 Mes, pH 7.0 buffered by Tris. Depending on the experimental variant, it was supplemented with 75 µM NED, 20 µM tetrandrine, or 20 µM verapamil. Bipartite Petri plates were used as experimental chambers. In the barrier dividing the plate into two compartments, a small aperture (about 1 mm width) was made, in which the thallus was inserted. The aperture was sealed with Vaseline.

### 4.2. Measurements in Nitellopsis obtusa

#### 4.2.1. Intracellular Microelectrode Recordings

Recordings of single plant cell (*N. obtusa*) membrane potential and APs were performed via the two-pair current clamp intracellular microelectrode technique in an aquatic environment as previously described in [54]: an intracellular glass microelectrode with a 1-µm tip diameter (filled with 3 M KCl) was inserted into the cell vacuole, and a reference glass electrode was immersed in the vicinity of the cell. The current (DC) was injected using separate extracellular Ag/AgCl electrodes.

After electrode impalement, a 1.5 h rest period followed, and then two APs were stimulated in 5-min intervals. APs were evoked by direct current increasing at a 0.02 µA/s ramp rate. Once AP excitation threshold potential (*E_th_*) was reached, the stimulating current was ceased. To examine the effect of each drug, only the area in the central compartment (0.5 mm of cell length) of the recording chamber was exposed to the drug solution under constant perfusion. The incubation time was 100 min. Next, two APs were evoked in a similar manner.

AP parameters were evaluated in accordance with [43]. Membrane resting potential (*RP*) was measured before each AP stimulation. *E_th_* of AP was determined as the membrane potential (MP) with a depolarization rate exceeding 60 mV/s. AP amplitude *A_th_* was evaluated from *E_th_* to AP peak potential, and AP amplitude *A_RP_* was evaluated from *RP* to AP peak potential. AP depolarization duration *t_dep_* was evaluated as a time needed for AP to depolarize from its half-amplitude *A_th_* value to the peak potential. AP repolarization duration *t_rep_* was evaluated as a time needed for AP to repolarize from the peak potential to its half-amplitude *A_th_* value. AP duration *t_1/2_* was calculated as the sum of *t_dep_* and *t_rep_*.

#### 4.2.2. Investigation of AP Conduction Velocity

The experiments were conducted as described in [27]. Briefly, an internodal cell of *N. obtusa* was placed in a custom-made plexiglass chamber consisting of 14 compartments filled with a solution (APW or a drug dissolved in APW). The compartments were electrically isolated with Vaseline. Two pairs of silver wires were used as extracellular electrodes. Each electrode was placed in a different compartment at a distance of at least 2 cm between adjacent wires. The signal was amplified and digitized using the PowerLab 8/30 system and LabChart 7 software (AD Instruments, Dunedin, New Zealand). Registration began immediately after 10-min incubation. APs were induced electrically (1 µA current stimulus lasting ~0.5 s) in a 10-min period using Stimulus Isolator A365 (WPI, Sarasota, FL, USA) unless the chemical compound induced spontaneous APs. In each case, the third AP of the induced AP series was used for analysis to ensure that the cells were in a stationary state. Conduction velocity was calculated from the time lag between two the same polarity peaks of a conducted AP and the distance between the corresponding recording electrodes. The registration lasted for 3–4 hours unless the cells became unexcitable sooner.

#### 4.2.3. Patch Clamping of Cytoplasmic Droplets

Cytoplasmic droplets were prepared from *N. obtusa* cells according to [50]. Briefly, an internodal cell was placed in a vertically positioned rubber tube filled with artificial cell sap (ACS). The bottom end of the cell was cut and the cytoplasm was allowed to flow into a bathing solution (ACS) for 30 min, where cytoplasmic droplets spontaneously formed. The droplets consisted of cytoplasm covered with the tonoplast. Patch clamp experiments were performed in a cell-attached configuration. Borosilicate micropipettes (WPI, Sarasota, FL, USA) were pulled by a P-1000 puller (Sutter Instrument, Co., Novato, CA, USA). The micropipettes were filled with a desired solution (ACS or a drug dissolved in ACS). The microelectrode was then connected to a signal registration system consisting of a MultiClamp 700B amplifier and Digidata 1440A ADC (Molecular Devices, San Jose, CA, USA) controlled by a PC via pClamp 10 software (Molecular Devices, San Jose, CA, USA). A chlorinated silver wire was used as a reference electrode. Sampling frequency was at least 10 kHz, filtered with a 1 kHz Bessel filter.

#### 4.2.4. Data Analysis

Data were analyzed using software pClamp 10, QuB [55], MicroCal OriginPro 2018 (OriginLab, Northampton, MA, USA), and the programming language R. Sample size *n* denotes cells or tonoplast patches. Statistical significance of differences was evaluated using the pair-sample Student’s *t*-test for individual cells if the samples were distributed according to normal distribution (Shapiro–Wilk test); otherwise, the Wilcoxon signed rank test was used. When the effects on different cells/tonoplast patches were being evaluated, the two-sample Student’s *t*-test was used (when the samples were distributed according to normal distribution; possible differences between sample variances were tested using the two-sample *t*-test for variance); otherwise, the Mann–Whitney test was employed. In all cases, the significance level was set to *p* = 0.05. The results are expressed as mean values ± SD. Linear regression curves are superimposed on single channel activity I/V curves (in all cases, *R*^2^ > 0.98).

### 4.3. Measurements in Marchantia polymorpha

#### 4.3.1. Intracellular Microelectrode Recordings

Electrically evoked action potentials in *Marchantia* were recorded with the microelectrode method. Microelectrodes were prepared from borosilicate glass tubes (WPI, Sarasota, FL, USA) pulled by a P-30 microelectrode puller (Sutter Instrument Co., Novato, CA, USA) and filled with 100 mM KCl. The Ag/AgCl reference electrode filled with 100 mM KCl contacted with the bath solution by a porous tip. The electrodes were connected to the amplifier FD 223 (WPI, Sarasota, FL, USA). The experiments were made inside a Faraday cage placed on an antivibration table (TMC Amatek Ultra Precision Technologies, Peabody, MA, USA). The data were collected by a Lab-Trax-4 device and registered on a PC hard drive by LabScribe 3 software (WPI, Sarasota, FL, USA). The sampling rate was 5 Hz. The microelectrode was inserted in the cell using a piezomicromanipulator Märzhäuser DC-3K (Märzhäuser Wetzlar GmbH & Co., Wetzlar, Germany).

#### 4.3.2. Stimuli

Plants were stimulated by rectangular electric stimuli (4 V, 1 s). Thin steel wires were used as stimulating electrodes. The stimulating electrodes were immersed in the solution in two compartments of the measuring chamber. The cathode was placed in the solution of the compartment in which the tested cell was located (this compartment was common for the microelectrode, reference electrode, and cathode). The anode was placed in the other compartment of the experimental chamber—on the opposite side of the electrically insulated barrier. 

#### 4.3.3. Data Analysis

Parameters describing membrane potential changes in *Marchantia* cells, e.g., membrane potential recorded before stimulation (RP), maximum value of membrane potential recorded during AP (*V_max_*), duration of AP measured in the half of the amplitude (*t_1/2_*), time from the moment of stimulation to reach the peak (*t_dep_*), time from the peak to half of repolarization (*t_rep_*), and amplitude of AP measured from resting potential (*A_RP_*) were analyzed in LabScribe 3 software. Statistical analyses were done in Sigma Stat 4.0 (Systat Software Inc., San Jose, CA, USA). Figures were prepared in Sigma Plot 9.0 (Systat Software Inc., San Jose, CA, USA), Grapher 9 (Golden software Inc., Golden, CO, USA), and Corel DRAW 12 (Corel Corporation, Ottawa, Canada).

#### 4.3.4. Patch Clamp Recordings and Analysis of *Marchantia polymorpha* Vacuolar Channels

Vacuoles of *Marchantia polymorpha* cells were isolated with a surgical method [28,56]. Briefly, fragments of thalli were plasmolyzed, cut with a sharp blade, and gradually deplasmolyzed in an isotonic solution, which caused release of isolated protoplasts and vacuoles. The recordings were carried out in the cytoplasm-out configuration. Single channel currents were recorded at a 60-mV test pulse lasting 20 s. The bath solution contained (in mM) 100 sodium gluconate, NaGlu, 0.1 CaCl_2_, 2 MgCl_2_, and 1 mM dithiothreitol, DTT, pH 7.2 buffered by 15 mM Hepes/Tris. The pipette contained (in mM) 100 NaGlu, 0.1 CaCl_2_, 2 MgCl_2_, and 1 mM DTT, pH 5.5 buffered with 15 mM Mes/Tris. The osmolarity of the solutions was adjusted by sorbitol to 500 mOsm in the bath medium and 550 mOsm in the pipette using a cryoscopic osmometer (Osmomat 030; Gonotec, Berlin, Germany). The patch pipettes made from borosilicate glass tubes (Kwik-Fill TW150-4; WPI, Sarasota, FL, USA) were pulled and fire-polished using a DMZ-Universal Puller (Zeitz-Instruments, Martinsried, Germany). The (Ag/AgCl) reference electrode filled with 100 mM KCl had contact with the bath medium via a ceramic porous tube. The measurements were carried out with an EPC10 amplifier (Heka Electronik, Lambrecht, Germany) working under Patchmaster software (Heka Electronik, Lambrecht, Germany). The sampling rate was 10 kHz and the samples were filtered with a 1 kHz low-pass filter. During the experiments, the solutions were exchanged by a peristaltic pump (ISM796B; Ismatec, Wertheim, Germany). The results are presented according to the convention of [57]. The amplitude histograms were made using GRAMS/AI 8.0 (Thermo Fisher Scientific, Waltham, MA, USA). The area under the Gaussian peak served to calculate the open probability of the channels.

## Figures and Tables

**Table 1:** Average values of resting potential (*RP*) and parameters describing the electrically evoked action potentials in the same internodal *Nitellopsis obtusa* cells in the control solution APW and after treatment with 300 µM verapamil, 100 µM tetrandrine, and 75 µM NED-19. Asterisks indicate a statistically significant difference (*p* < 0.05).

	APW	Verapamil 300 µM	APW	NED-19 75 µM	APW	Tetrandrine 100 µM
RP (mV)	−210 ± 11 (*n* = 7)	−140 ± 62 (*n* = 7)*	201 ± 15 (*n* = 7)	−155 ± 33 (*n* = 7)*	−203 ± 21 (*n* = 6)	−165 ± 44 (*n* = 6)*
E_th_ (mV)	−101 ± 12 (*n* = 7)	−73 ± 35 (*n* = 7)*	−108 ± 9 (*n* = 7)	−81 ± 20 (*n* = 7)*	−78 ± 18 (*n* = 6)	−80 ± 17 (*n* = 6)
V_max_ (mV)	36 ± 14 (*n* = 7)	37 ± 24 (*n* = 7)	35 ± 10 (*n* = 7)	27 ± 25 (*n* = 7)	30 ± 4 (*n* = 6)	32 ± 6 (*n* = 6)
A_th_ (mV)	137 ± 21 (*n* = 7)	110 ± 52 (*n* = 7)	143 ± 10 (*n* = 7)	107 ± 31 (*n* = 7)*	107 ± 18 (*n* = 6)	112 ± 18 (*n* = 6)
A_RP_ (mV)	254 ± 14 (*n* = 7)	178 ± 79 (*n* = 7)*	231 ± 17 (*n* = 7)	181 ± 33 (*n* = 7)*	233 ± 21 (*n* = 6)	197 ± 42 (*n* = 6)
t_dep_ (s)	0.8 ± 0.1 (*n* = 7)	1.6 ± 0.7 (*n* = 7)*	0.9 ± 0.1 (*n* = 7)	1 ± 0.2 (*n* = 7)	1.3 ± 0.2 (*n* = 6)	1.9 ± 0.3 (*n* = 6)*
t_rep_ (s)	2.6 ± 0.7 (*n* = 7)	8 ± 8.6 (*n* = 7)*	3.5 ± 1.2 (*n* = 7)	6 ± 3.4 (*n* = 7)	2.6 ± 0.8 (*n* = 6)	8.2 ± 3.9 (*n* = 6)*
t_1/2_ (s)	3.5 ± 0.8 (*n* = 7)	9.6 ± 9.2 (*n* = 7)*	4.4 ± 1.2 (*n* = 7)	6.9 ± 3.4 (*n* = 7)*	3.9 ± 0.9 (*n* = 6)	10.1 ± 4 (*n* = 6)*

**Table 2:** Average values of resting potential (*RP*) and parameters describing the electrically evoked action potentials in *Marchantia polymorpha* thalli in the control plants and after the treatment with 20 µM verapamil, 20 µM tetrandrine, and 75 µM NED-19. Asterisks indicate statistically significant difference (*p* < 0.05).

	Standard	20 µM Verapamil	20 µM Tetrandrine	75 µM NED-19 (short)	75 µM NED-19 (long)
RP (mV)	−187± 12 (*n* = 16)	−163 ± 10 (*n* = 10)*	−130 ± 12 (*n* = 8)*	−172 ± 17 (*n* = 8)*	−165 ± 21 (*n* = 6)*
V_max_ (mV)	−47 ± 23 (*n* = 16)	−98 ± 34 (*n*=6)*	−58 ± 18 (*n*=8)	−46 ± 15 (*n* = 8)	−46 ± 13 (*n* = 6)
A_RP_ (mV)	139 ± 19 (*n* = 16)	70 ± 33 (*n* = 6)*	72 ± 13 (*n* = 8)*	126 ± 9 (*n* = 8)*	119 ± 11 (*n* = 6)*
t_dep_ (s)	4.8 ± 1.6 (*n* = 16)	5.5 ± 1.7 (*n* = 6)	5.9 ± 1.1 (*n* = 7)	5.9 ± 1.6 (*n* = 8)	6.1 ± 1.7 (*n* = 6)
t_rep_ (s)	19.9 ± 6.5 (*n* = 16)	13.4 ± 5.7 (*n* = 6)	169.8 ± 76.2 (*n* = 7)*	30.7 ± 13.0 (*n* = 8)*	184.3 ± 46.2 (*n* = 6)*
t_1/2_ (s)	24.7 ± 7.3 (*n* = 16)	18.9 ±6.9 (*n* = 6)	184.5 ± 74.8 (*n* = 8)*	36.6 ± 12.1 (*n* = 8)*	190.4 ± 45.4 (*n* = 6)*

## Data Availability

The data presented in this study are available on request from the corresponding authors. The data are not publicly available due to privacy.
